# Bivalent Inhibitor Development for a Dynamic Epigenetic Tandem Reader Protein Using a Combinatorial Histone Code

**DOI:** 10.64898/2026.10.09.758058

**Published:** 2026-10-16

**Authors:** Joseph E. McPherson, Molly S. Sneddon, Richard K. Begyinah, Kesavan Babu, Kerstin E. Peterson, Ryan F. Friess, Thomas G. Smith, Natalia Y. Tretyakova, Marcus Fischer, William C.K. Pomerantz

## Abstract

Epigenetic readers are a class of proteins that recognize specific chemical modifications on histones to differentially regulate gene expression. Aberrant expression of readers can drive the progression of many diseases and cancers, making this protein class viable therapeutic targets. Understanding the landscape of histone modifications recognized by reader domains, and the structural mechanisms that drive these molecular recognition events, is crucial for guiding the design of pharmaceutically relevant chemical probes. BPTF contains a plant homeodomain (PHD) finger and tandem bromodomain (BRD). Here, we expand upon previous studies that found the BPTF BRD can engage acetyllysine modifications on the N-terminus of histone H3. We uncover H3K4me _3_ K14ac as the specific modifications recognized by the PHD-BRD cassette, which is confirmed by crystallography. Using orthogonal methods, our work explores how these modifications can be read cooperatively, including in *cis.* We report the first experimental evidence proposing a ligand-induced conformational change between the two reader domains of BPTF, supporting previous computational approaches that have predicted a macromolecular rearrangement. We leverage these structural insights to rationally design a peptide-small molecule conjugate **MJ-1** that inhibits both domains of BPTF and overcomes ligandability challenges associated with PHD-finger-containing proteins. The results presented here elucidate the molecular basis of the bivalent recognition of multiply modified histone H3 by BPTF and provide structural insight for developing compounds targeting the PHD finger of BPTF for therapeutic applications.

